# Sterile endophthalmitis after intravitreal triamcinolone acetonide injection: A case report series

**DOI:** 10.1016/j.amsu.2022.103537

**Published:** 2022-03-28

**Authors:** Ahmed Mahjoub, Nadia Ben Abdesslem, Atf Ben Abderrazek, Nesrine Zaafrane, Anis Mahjoub, Hichem Aoun, Ahmed Jabri, Fethi Krifa, Mohamed Ghorbel, Hachemi Mahjoub

**Affiliations:** a Department of Ophthalmology, Farhat Hached University Hospital of Sousse, Tunisia; b Faculty of Medicine of Sousse, University of Sousse, Tunisia

**Keywords:** Case report series, Intravitreal injection, Triamcinolone acetonide, Sterile endophthalmitis, TA, Triamcinolone acetonide, IVTA, Intra vitreal triamcinolone acetonide, BCVA, Best corrected visual acuity, IOP, Intraocular pressure, OCT, Optical coherence tomography

## Abstract

Corticosteroids have proven their effectiveness in the treatment of cystoid macular edema. Especially after an intravitreal injection. Triamcinolone acetonide is the most commonly used in the treatment of macular edema. Noninfectious endophthalmitis is a form of endophthalmitis that can occur in the absence of a defined germ after an intravitreal injection of triamcinolone acetonide.

We report here the case of three diabetic patients, who presented with visual blur, three days after an intra vitreal triamcinolone acetonide injection performed in the right eye.

The vitreous inflammation resolved spontaneously in the first two cases after three weeks, and after four weeks for the third. The diagnosis of sterile endophthalmitis was made in view of the spontaneous resolution of the inflammation without the use of intravitreal injection of antibiotics and/or vitreoretinal surgery.

## Highlights

• Describe the development of sterile endophthalmitis after an intravitreal injection of triamcinolone acetonide in diabetic patients with macular edema.
• Enhance the differences between infections endophthalmitis and sterile endophthalmitis.
• Report our ophthalmology team's management in front of a sterile endophthalmitis in comparison to literature.

## 1 Introduction and importance

Corticosteroids have been proving their effectiveness in the treatment of cystoid macular edema due to their anti-angiogenic, anti-edematous and anti-inflammatory properties [1].

Triamcinolone acetonide (TA) is a synthetic corticosteroid, from the glucocorticoid family. It is the most commonly used in the treatment of macular edema. TA has five times the anti-inflammatory potency of hydrocortisone [2].

It is presented as a white-colored, crystalline powder, insoluble in water. This can explain its prolonged duration of action. Its therapeutic effects last for about three months, especially after an intravitreal injection of 4 mg [2].

The vitreous cavity presents indeed a reservoir where high concentrations of corticosteroids can be obtained, with minimal systemic adverse effects [3].

Nevertheless, this route is not without adverse-effects. One of the rarest adverse-effects of intravitreal triamcinolone injection is endophthalmitis.

Considered as a serious intraocular complication, three variants of endophthalmitis that may result from intravitreal injection of triamcinolone can be defined: Endophthalmitis secondary to germ infection, pseudo endophthalmitis secondary to precipitation of triamcinolone crystals, and sterile endophthalmitis defined by a rapid inflammatory reaction in the absence of a defined germ [4].

This case report has been reported in line with the SCARE criteria [5].

## 2 Case presentation

We report here the case of three diabetic patients, who presented to emergency department with visual blur, three days after an intra vitreal triamcinolone acetonide injection (IVTA) performed in the right eye. All three patients complained of ocular discomfort and decreased visual acuity in the eye where they had the injection.

A complete ophthalmologic examination, as well as visual acuity measurement and intraocular pressure measurement and ocular ultrasound were performed. Fundus examination and optical coherence tomography could not be performed because of the vitreous haze.

**Fig. 1:**
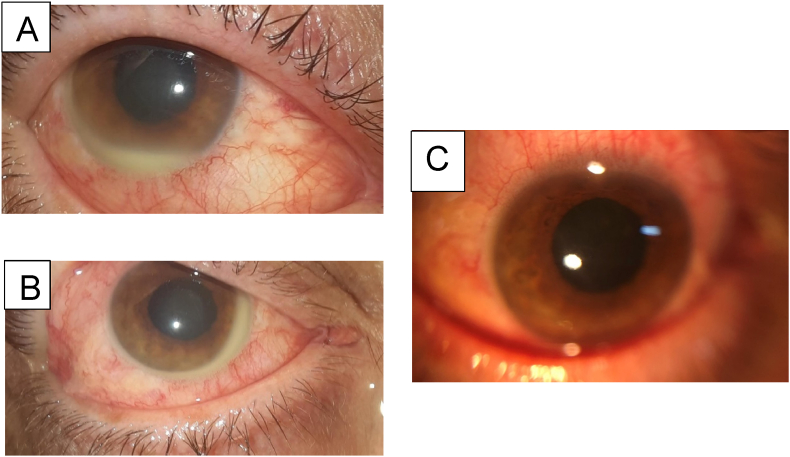
**A, B**: Clinical photograph of the right eye taken at time of presentation, 3 days after the IVTA exhibiting a gravity dependent 3mm hypopyon. The visual acuity was hand motion, the cornea was clear, the anterior chamber an inflammatory reaction with a cyclitic membrane. Note the minimal conjunctival injection and the lack of fibrin despite clear evidence of vitritis **C:** Anterior segment photo taken 15 days after the IVTA exhibiting the hypopyon resolution.
Case 2 Right eye: BCVA: hand movement, normal IOP, 4mm Hypopyon (Fig. 2), anterior chamber cells, Flare to 3, triamcinolone vitreous condensation (Fig. 3), vitreous Haze to 3 +, An inaccessible Fundus

Case 1 Right eye:Best corrected visual acuity (BCVA) = hand movement, normal intraocular pressure (IOP), 3mm Hypopyon (Fig. 1), anterior chamber cells 2 +, Flare to 3 + with the presence of cyclitic membrane, vitreous Haze to 3 +, An inaccessible Fundus

**Fig. 2:**
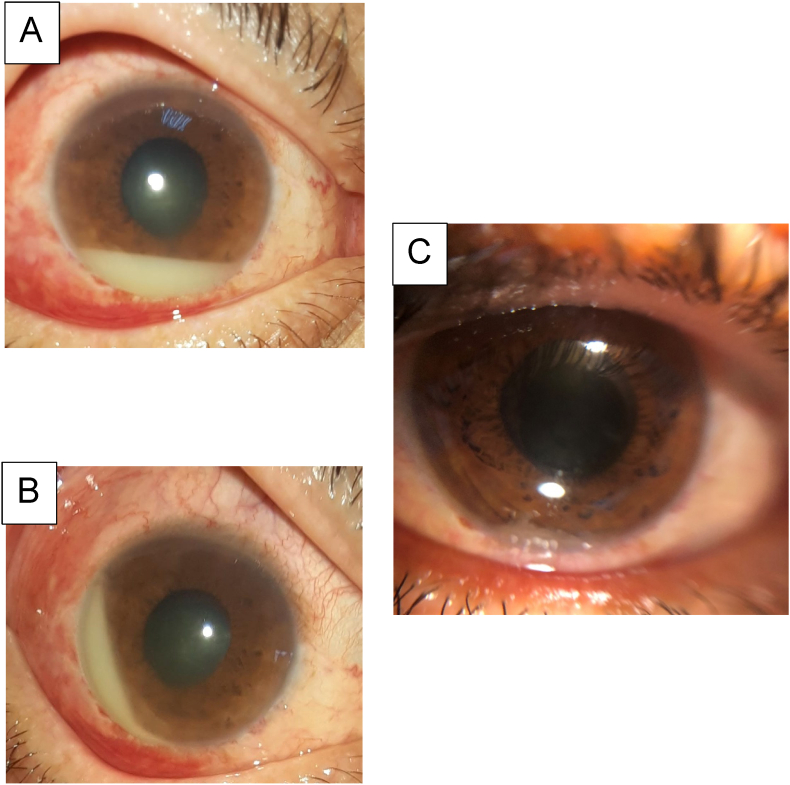
**A, B**: Anterior segment photo taken 3 days after the IVTA exhibiting the 4mm gravity-dependent hypopyon The visual acuity was hand motion, the cornea was clear, the anterior chamber an inflammatory reaction. Note the minimal conjunctival injection and the lack of fibrin despite clear evidence of vitritis. **C:** Anterior segment photo taken 15 days after the IVTA exhibiting the resolution of hypopyon.

**Fig. 3:**
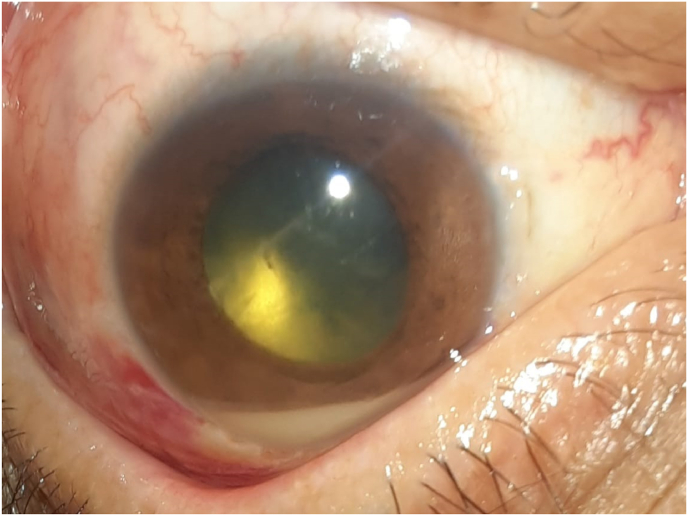
Anterior segment photo showing vitreous condensation of triamcinolone.
Case 3 Right eye: BCVA: counting fingers, normal IOP, anterior chamber cells, 1mm Hypopyon (Fig. 4), anterior chamber cells to 1 cross, Flare to 1+, A vitreous Haze at 1 +, A blurred Fundus

**Fig. 4:**
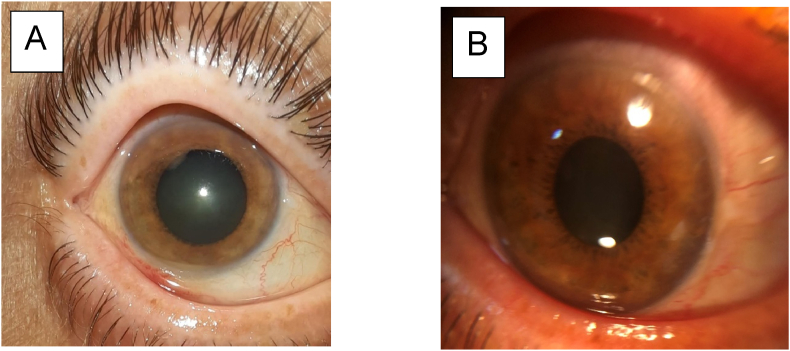
**A** Anterior segment photo taken 3 days after the IVTA exhibiting the 1mm gravity dependent hypopyon. Note the absence of conjunctival injection. And the minimal inflammatory reaction despite the vitritis. **B** Anterior segment photo taken 15 days after the IVTA exhibiting the resolution of hypopyon.

Each patient received an intravitreal injection of 4mg of triamcinolone. Each performed in the operating room. After topical disinfection with povidone-iodine, a sterile field and wire lid speculum were applied. A local anesthesia with Oxybuprocaine 0.4% and a local antibiotic therapy at the end of the procedure were applied. The injection was performed by a 30 gauge syringe, 4mm from the limbus. The suspension was shaken vigorously to ensure the correct concentration. The supernatant was not removed.

All three patients received local treatment consisting of antibiotics, corticosteroids and cycloplegics.

**Fig. 5:**
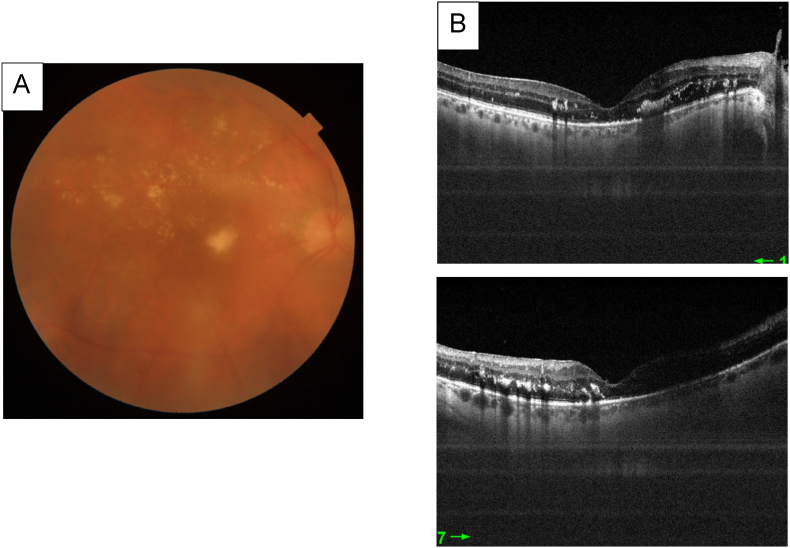
**A** Fundus photography taken 15 days after the IVTA showing severe non proliferative diabetic retinopathy and perifoveal exudates with a complete resorption of triamcinolone. **B** Macular Swept source OCT showing a flat macula with perifoveal exudates.
Case 2 Right eye: BCVA = 20/200 normal IOP, anterior chamber cell at 0.5 +, A vitreous haze at 1 +, Fundoscopy examination showing severe non proliferative diabetic retinopathy (Fig. 6)

2 weeks after IVTA, the ophthalmologic examination showed:

Case 1 Right eye: BCVA = 20/200, normal IOP, anterior chamber cell at 0.5 +, A vitreous haze at 0.5 +, Fundoscopy examination showing severe non proliferative diabetic retinopathy (Fig. 5)

**Fig. 6:**
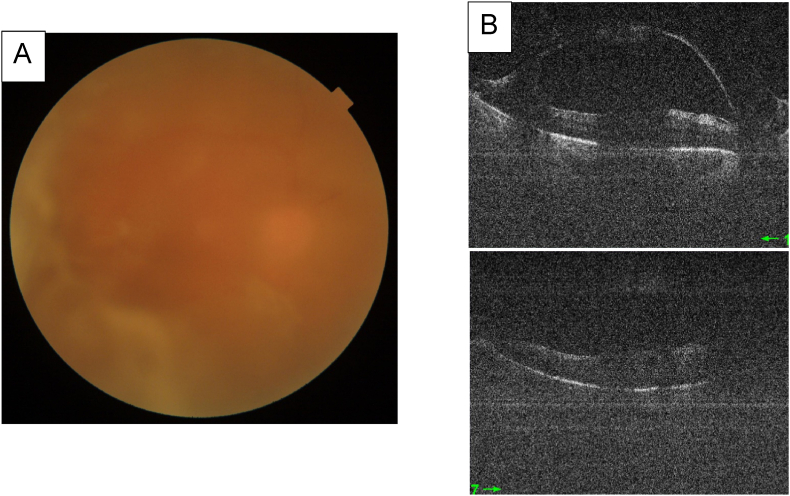
**A** Fundus photography taken showing blurry fundoscopy with triamcinolone loaded vitreous **B** Macular Swept source OCT showing posterior vitreous detachment. Mediocre signal is due to triamcinolone loaded vitreous. The macula appears to be flat.
Case 3 Right eye: BCVA = 20/200 normal IOP, no chamber cell inflammation , A vitreous haze at 0.5 +, Vitreous condensation of triamcinolone localized in inferior temporal, Fundoscopy examination showing severe non proliferative diabetic retinopathy (Fig. 7)

**Fig. 7:**
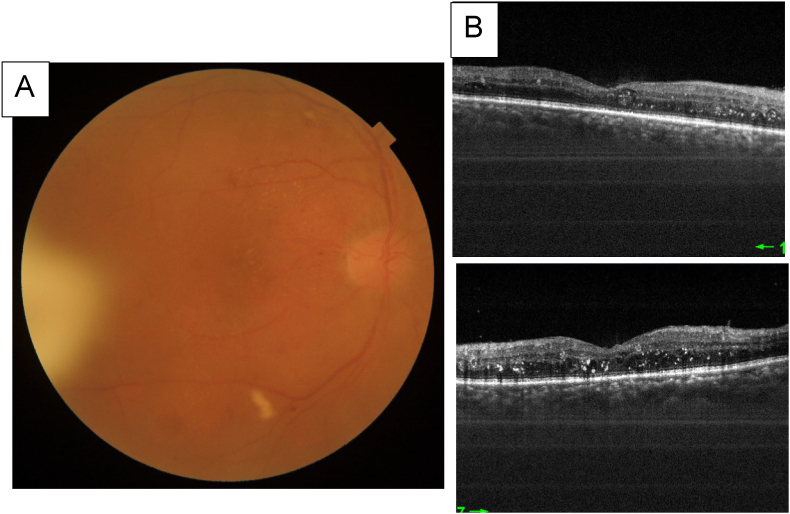
**A**: Photo Fundus taken showing a vitreous condensation of triamcinolone localized in inferior temporal **B:** Swept source OCT showing macular exudates, with a flat macula.

The vitreous inflammation resolved spontaneously in the first two cases after three weeks, and after four weeks for the third. The diagnosis of sterile endophthalmitis was made in view of the spontaneous resolution of the inflammation without the use of intravitreal injection of antibiotics and/or vitreoretinal surgery.

This case report has been reported in line with the SCARE criteria [5].

## 3 Clinical discussion

Sterile endophthalmitis following IVTA is relatively uncommon with an incidence rate generally reported as being between 0.1% and 7.3% [6,7].

It appears more frequently in patients who underwent cataract surgery or vitrectomy [8].

While infectious endophthalmitis is expected to appear more than three days after IVTA, sterile endophthalmitis usually appears within 1–3 days after injection.

It is characterized by less marked vision loss, mild to moderate functional signs and a gravity-dependent chalk white hypopyon. Patients complain more of a visual discomfort rather than a real pain. Although pain can be attenuated by the anti-inflammatory IVTA effect [9].

The strongest argument in favor of the sterile origin of endophthalmitis remains the spontaneous resolution without the use of intravitreal antibiotic treatment.

In opposition to infectious endophthalmitis, patients with sterile endophthalmitis seem to have a better ocular prognosis. The visual acuity deterioration is usually a result of the underlying disease [10].

Nevertheless, serious complications such as vitreous hemorrhage and retinal detachment have been described in litterature [11].

Our three patients only developed minor pain. They had minimal conjunctival injection. Two of them had a gravity-dependent chalk white hypopyon that shifted with head position. The three of them returned to baseline visual function within three weeks.

Unfortunately, we could not provide any Gram stain or microbiological culture of the anterior chamber or vitreous tap to prove the absence of a germ. We mainly chose to observe rather than treat according to information from literature review that reinforce the hypothesis of the sterile origin.

The pathogenesis of noninfectious endophthalmitis following IVTA remains controversial.

An inflammatory reaction to preservatives has been the leading hypothesis for the sterile inflammatory response following IVTA. Numerous studies have suggested the involvement of benzyl alcohol used in the preparation of triamcinolone in sterile endophthalmitis [12,13].

Nevertheless, other studies have refuted this theory, reporting the development of sterile endophthalmitis even after filtering the TA and removing the benzyl alcohol [14]. Lam et al. demonstrated that sterile endophthalmitis can occur even with preservative-free triamcinolone [15].

A possible link between the occurrence of sterile endophthalmitis and the size and concentration of the particles used in the triamcinolone formulation has been proposed by some authors. Low-weight particles would then be more incriminated [16].

Other theories suggest that endotoxins in TA formulations can cause sterile endophthalmitis following IVTA. Animal models have proved that a single intravitreal injection of 0.01–500μg of bacterial endotoxin can be responsible for dose-dependent endophthalmitis [17].

However, other studies reported that vials from the same lots as those patients developing sterile endophthalmitis were negative for endotoxin [18].

There are adverse effects related to the use of corticosteroids and others related to the injection itself.

Elevation of intraocular pressure and cataract formation are secondary to corticosteroid use.

In contrast, intravitreal injection could result in infectious endophthalmitis, sterile endophthalmitis, and retinal detachment [7]. The increased awareness for those ocular complications is tempering the enthusiasm for the off-label use of IVTA for vitreoretinal pathologies.

## 4 Conclusion

In conclusion, sterile endophthalmitis should be evoked in front of every ocular inflammatory reaction involving decreased vision, moderate functional signs, and a gravity-dependent hypopyon occurring within 3 days of an IVTA.

Local treatment with corticosteroids, antibiotics, and cycloplegics could be attempted as first-line treatment. Patients who do not need further surgery can avoid unnecessary intervention and clear their inflammation spontaneously within three weeks. When in doubt, the ophthalmologist must reconsider the diagnosis and treat as an infectious endophthalmitis because of the poor prognosis of this complication.

## Ethical approval

We further confirm that any aspect of the work covered in this manuscript that has involved human patients has been conducted with the ethical approval of all relevant bodies and that such approvals are acknowledged within the manuscript. IRB approval was obtained (required for studies and series of 3 or more cases) Written consent to publish potentially identifying information, such as details or the case and photographs, was obtained from the patient(s) or their legal guardian(s).

## Funding

No funding or grant support.

## Author contribution

Ahmed Mahjoub: writing the paper, Nadia Ben Abdesslem: data analysis, Atf Ben Abderrazek: writing the paper, Nesrine Zaafrane: data collecting, Anis Mahjoub: study concept, Hichem Aoun: study design, Ahmed Jabri: data interpretation, Fathi Krifa: correcting the final paper, Mohamed Ghorbel: correcting the final paper, Hachemi Mahjoub: correcting the final paper.

## Trail registry number

Name of the registry:

Unique Identifying number or registration ID:

Hyperlink to your specific registration (must be publicly accessible and will be checked):

## Guarantor

Atf ben abderrazek, resident.

## Authorship

All authors attest that they meet the current ICMJE criteria for Authorship.

## Provenance and peer review

Not commissioned, externally peer reviewed.

## Patient consent

Written informed consent was obtained from the patients for publication of this case report and accompanying images. A copy of the written consent is available for review by the Editor-in-Chief of this journal on request”.

## Declaration of competing interest

The following authors have no financial disclosures: The following authors have no financial disclosures: (Ahmed Mahjoub, Nadia Ben Abdessalem, Atf Ben Abderrazek, Nesrine Zaafrane, Anis Mahjoub, Ilhem Sellem, Ahmed Jabri, Fethi Krifa, Mohamed Ghorbel, Hachemi Mahjoub).
